# Anti-Mullerian hormone is linked to the type of early pregnancy loss in idiopathic recurrent miscarriage: a retrospective cohort study

**DOI:** 10.1186/s12958-017-0278-x

**Published:** 2017-08-02

**Authors:** Sophie Pils, Natalia Stepien, Christine Kurz, Kazem Nouri, Regina Promberger, Johannes Ott

**Affiliations:** 1 Department of Gynecological Endocrinology and Reproductive Medicine, Medical University of Vienna, Waehringer Guertel 18-20, 1090 Vienna, Austria; 2 Department of Obstetrics and Gynecology, Saint John of God Hospital Eisenstadt, Johannes von Gott-Platz 1, 7000 Eisenstadt, Austria; 3 Clinical Division of Gynecological Endocrinology and Reproductive Medicine, Medical University of Vienna, Waehringer Guertel 18-20, A-1090 Vienna, Austria

**Keywords:** Recurrent early pregnancy loss, Recurrent miscarriage, Ovarian reserve, Anti-Mullerian hormone, Follicle stimulating hormone

## Abstract

We correlated Anti-Mullerian hormone (AMH) levels and other parameters for ovarian reserve to the gestational age at the time of pregnancy loss in women with idiopathic recurrent miscarriage. In a retrospective study, 79 patients had suffered a total of 266 miscarriages. When comparing women with an “unembryonic” to those with an “embryonic” most recent miscarriage, there was no difference in median age (36.3 years, IQR 31.6–40.1 versus 34.2 years, IQR 29.9–38.0; *p* = 0.303) but in median AMH levels (0.7, IQR 0.2–18, versus median 1.8, IQR 1.3–3.3, respectively, *p* = 0.044) and in the rate of patients with an AMH ≤ 1 ng/mL (23/37, 62.2%, versus 8/42, 19%; *p* < 0.001). Thus, AMH might add to the diagnostic process in recurrent miscarriage in the future.

## Introduction

A link between decreased ovarian reserve parameters and recurrent miscarriage (RM) has been demonstrated [1, 2]. This makes an association of RM with decreased oocyte quality likely. Indeed, studies have identified increased rates of chromosomal abnormalities in embryos derived from couples with RM [3].

The rate of euploid miscarriages is positively correlated with estimated gestational age at pregnancy loss [4]. The same holds true when comparing clinical to preclinical miscarriages [5]. Thus, ovarian reserve markers and gestational age at miscarriage in women with idiopathic RM (IRM) might also be linked to each other and knowledge about an association like this could help in clinical counselling. The aims of this study were to correlate markers of reproductive age with gestational age at and the type of miscarriage in women with IRM.

## Methods

Seventy-nine women with IRM who had undergone a complete diagnostic evaluation at the Medical University of Vienna (MUW) from January 2006 to January 2016 were retrospectively included. RM was defined based on a documented history of at least three spontaneous, consecutive miscarriages before 20 weeks’ gestation (biochemical pregnancies excluded). In absence of uterine malformations, thrombophilic defects, abnormalities of maternal/paternal karyotype, antiphospholipid syndrome, diabetes mellitus, and any hormonal dysfunctions, IRM was defined [2]. The study was approved by the Institutional Review Board (IRB number 2088/2016). Data in this retrospective study was anonymized, so informed consent was waived.

We examined basal serum levels of follicle stimulating hormone (FSH), luteinizing hormone (LH), AMH, and estradiol that had been assessed in the course of routine diagnostic evaluation of RM and had been determined in the central laboratory of the MUW, using radioimmunoassays (estradiol: Autodelfia; Wallac Oy, Turku, Finland; LH: Autodelfia; Wallac Oy, Turku, Finland; FSH: Enzymun ES700; Boehringer Mannheim, Mannheim, Germany; and AMH: DSL Active MIS/AMH assay; Beckman Coulter Inc., Brea, USA). The AMH cut-off for poor ovarian reserve was defined as ≤1 ng/mL [6]. Other parameters were: patients’ age, smoking status and body mass index (BMI); calculated gestational age at previous pregnancy losses; the type of previous pregnancy losses as defined by the ESHRE Special Interest Group in early pregnancy [7]: “early miscarriages” (<10 weeks of gestation and/or no cardiac activity) versus “fetal miscarriage” (≥10 weeks of gestation and cardiac activity), and “without an embryo” (i.e., anembryonic/empty sac miscarriages + yolk sac miscarriages) versus “embryonic/fetal miscarriages”; and whether patients had achieved further pregnancies and their outcome (further miscarriages and/or live births >week 23 + 0).

Statistical analyses were performed with the SPSS software package, version 17.0 (SPSS, Chicago). Nominal variables are reported as numbers and frequencies, and continuous variables with median and range. Differences between groups were tested using the chi-square or Fisher’s exact test (where appropriate) for nominal variables. Multivariate binary logistic regression models were used to test the influence of ovarian reserve parameters on types of miscarriages and regression co-efficients ß and their standard deviations are provided. Correlations between numeric parameters were tested using bivariate Spearman tests including hypothesis tests for significance of the correlation coefficient. Differences were considered significant if *p <* 0.05.

## Results

Patients had suffered from 266 miscarriages (3, 4, and 5 consecutive miscarriages in 54, 21, and 4 cases, respectively) at a median gestational age of 8 completed weeks (IQR, 7–9). Of these, 213 (82.3%) had been “early” and 47 (17.7%) had had “no embryo”. Median BMI was 23.4 kg/m^2^ (IQR, 21.5–25.7). The median time interval between the most recent miscarriage and diagnostic evaluation was 5 months (IQR, 4–7). Table 1 shows details about hormonal testing.

**Table 1:** Parameters for ovarian reserve in the whole study population, as well as according to gestational age at and the type of the most recent miscarriage – results of the logistic regression models

	*Complete study population*	*Early miscarriages*	*Fetal miscarriages*	*ß (standard deviation of ß)*	*p*	*Miscarriages without an embryo*	*Embryonic/fetal miscarriages*	*ß (standard deviation of ß)*	*p*
N ^#^	79	62 (78.5)	17 (21.5)		-	37 (46.8)	42 (53.2)		
Age (years) *	35.2 (30.9; 39.1)	35.1 (31.3–39.5)	36.9 (29.0–38.3)	0.015 (0.051)	0.765	36.3 (31.6–40.1)	34.2 (29.9–38.0)	0.054 (0.042)	0.204
BMI (kg/m^2^)	24.2 (21.7–26.4)	24.0 (21.7–26.2)	25.4 (22.1–27.4)	−0.042 (0.073)	0.564	23.6 (21.6–25.7)	25.1 (22.4–27.0)	−0.083 (0.062)	0.182
Smoking ^#^	11 (13.9)	5 (8.1)	6 (35.3)	−2.156 (0.757)	*0.004*	3 (8.1)	8 (19.0)	−1.382 (0.786)	0.079
LH (IU/L) *	5.4 (3.9; 8.1)	5.3 (3.8–8.1)	6.7 (4.2–8.1)	−0.065 (0.092)	0.481	5.4 (4.0–8.3)	5.6 (3.4–7.8)	0.032 (0.075)	0.670
FSH (IU/L) *	5.3 (3.4; 7.2)	5.8 (3.1–7.3)	5.0 (4.0–6.9)	0.014 (0.104)	0.896	4.5 (2.9–7.4)	5.4 (3.8–6.9)	−0.079 (0.087)	0.364
Estradiol (pg/mL) *	98 (49; 144)	97 (50–141)	112 (36–179)	0.001 (0.003)	0.687	85 (47–159)	99 (60–131)	0.000 (0.002)	0.967
AMH (ng/mL) *	1.5 (0.6; 2.5)	1.4 (0.5–2.4)	2.0 (0.8–3.4)	−0.145 (0.133)	0.278	0.7 (0.2–1.8)	1.8 (1.3–3.3)	−0.298 (0.155)	*0.045*

Data are presented as *median (interquartile range) for numerical parameters or as ^#^numbers (frequencies) for categorical parameters

^*^ Statistically significant results are presented in italic

When testing ovarian reserve parameters and their correlations with the median gestational age at miscarriages, patient age (*r =* −0.122; *p =* 0.283), LH (*r =* 0.102; *p =* 0.371), FSH (*r =* 0.134; *p =* 0.240), and estradiol (*r =* 0.045; *p =* 0.695) did not show significant results, while AMH revealed a significant positive correlation (*r =* 0.237; *p =* 0.035). Similar results were found for gestational age at the most recent miscarriage, with *p*-values >0.05 for age (*r =* −0.200; *p =* 0.078), LH (*r =* 0.025; *p =* 0.826), FSH (*r =* −0.009; *p =* 0.935), and estradiol (*r =* 0.157; *p =* 0.166), and, again, a significant positive correlation for AMH (*r =* 0.316; *p =* 0.005).

When focusing only on the most recent miscarriage (Table 1), significant differences were neither found for serologic ovarian reserve parameters between women with “early” (62/79, 78.5%) and “fetal miscarriages” (17/79, 21.5%) nor median age (35.1 years, IQR 31.3–39.5 versus 36.9, IQR 29.0–38.3; *p* = 0.765). However, among women after a most recent “fetal miscarriage”, significantly more smokers were found (*p =* 0.004). Women whose most recent miscarriage was classified as “without an embryo” (37/79, 46.8%) revealed significantly lower AMH levels than women whose most recent miscarriage was classified as embryonic/fetal miscarriage (42/79, 53.2%; median 0.7, IQR 0.2–18, versus median 1.8, IQR 1.3–3.3, respectively, *p =* 0.045) while patient age and the other ovarian reserve parameters showed no significant differences.

Moreover, 26/62 women (41.9%) whose most recent miscarriage was “early” showed an AMH level ≤ 1 ng/mL compared to 5/17 women (29.4%) whose most recent miscarriage “fetal” (*p =* 0.411). In 23/37 women (62.2%) whose most recent miscarriage was classified as “without an embryo,” AMH levels ≤1 ng/mL were found compared to 8/42 women (19.0%) whose most recent miscarriage was “embryonic/fetal” (*p <* 0.001).

## Discussion

It has recently been demonstrated that women with RM have lower AMH levels [1], which were even more pronounced in cases of IRM [2]. The present retrospective study suggests that in women with IRM, this parameter was positively correlated with the mean gestational age at miscarriage per patient, while patient age was not. One might argue that the hypothesis tests for significance of the correlation coefficient might not be highly reliable. However, these results were in line with other findings in our data set: 62% of women whose most recent miscarriage was classified as “without an embryo” demonstrated low AMH levels ≤1 ng/mL compared to 19% in women whose last miscarriage was classified as “embryonic/fetal.” This is in line with the result of the multivariate analysis (Table 1) showing significantly lower median AMH levels in women after a most recent miscarriage “without an embryo”. The association of diminished ovarian reserve with very early pregnancy losses would be confirmed by the assumed link between a higher biological age and a trend toward chromosomal abnormalities that lead to early miscarriage. It has already been shown that euploid miscarriages were more likely clinical (≥6 weeks) than preclinical (<6 weeks) [5]. Moreover, embryonic poles that were two weeks smaller than expected were associated more often with aneuploidy [3], which would be somewhat in line with our results. Likewise, a small crown rump-length measurement at the first assessment was associated with aneuploidy in another study [8]. However, the literature on this topic is quite controversial [9, 10] and also includes reports about similar euploidy and aneuploidy rates in empty gestational sacs [3], as well as a higher frequency of genetic abnormalities in the embryonic period than in the pre-embryonic or fetal stages [11]. Moreover, ovarian reserve parameters including the antral follicular count have not been proven to predict miscarriage rates, for example in a study on patients undergoing unstimulated therapeutic donor insemination [12] which definitely is a contradiction to the results of our data set. However, this difference could also be due to the fact that we dealt with women suffering from recurrent miscarriage and not infertility.

Although it might not be the case that anembryonic/empty sac miscarriages or yolk sac miscarriages are those with the highest aneuploidy rates, our data clearly suggest that a higher biological age, as indicated by lower AMH levels, was associated with very early types of pregnancy losses. Although we cannot provide data on genetic abnormalities in our patient population, which should be seen as a limitation as the retrospective approach, we believe that these new insights should help counsel women with IRM in the future. It is likely that ovarian reserve parameters will be implemented as routine parameters in the evaluation process of RM one day.

It will be of high clinical impact to evaluate whether AMH might predict the chances of future miscarriages or live births after IRM. However, we cannot provide reliable data on these issue in our current data set due to its retrospective design and the small sample size which must be considered general study limitations. This obviously limits this study’s informative value.

It must be noted that FSH, the most widely performed test for ovarian reserve [13], was not of influence in the regression models (Table 1). This is somehow in line with previous studies on ovarian reserve testing that suggested only a minor [1] or no influence [2] of FSH in RM. Another interesting finding that smoking was associated with “fetal” and not “early” most recent miscarriages. It has been already suggested that - apart from its detrimental effect on fertility - smoking was associated with an increased risk for miscarriages which might be due to deficiency of the developing placenta and consecutively restricted embryonic and fetal growth [14]. Thus, it seems reasonable that smoking could lead to fetal instead of early miscarriages.

## Conclusion

To the best of our knowledge, this is the first study to compare markers of ovarian reserve with age at pregnancy loss in women with RM. Our data suggest that AMH might be a valuable parameter in addition to chronological age in the future. Larger prospective trials are warranted to prove the finding that lower AMH levels were associated with earlier gestational age at miscarriage and to further evaluate the value of AMH in the diagnostic process of RM.

## Abbreviations

AMH: Anti-Mullerian hormone
FSH: follicle stimulating hormone
IRM: idiopathic RM
LH: luteinizing hormone
RM: recurrent miscarriage
TSH: Thyroid stimulating hormone
